# Back to nature: henna extracts from nanotech to environmental biotechnology – a review

**DOI:** 10.5114/bta.2023.132776

**Published:** 2023-12-21

**Authors:** Desouky A.M. Abd-El-Haleem

**Affiliations:** Environmental Biotechnology Department, Genetic Engineering and Biotechnology Research Institute, City of Scientific Research and Technological Applications, Burgelarb, Alexandria, Egypt

**Keywords:** henna extracts, biotechnology, nanotechnology, renewable energy, pollution control, textile fabrics

## Abstract

The Lythraceae family includes henna (*Lawsonia inermis*), which thrives in subtropical and tropical climates. One of its many and long-standing uses is in cosmetics as a pigment to color hair and nails. Additionally, it serves as a disinfectant against microbiological infections and has traditional applications in the textile industry, specifically for coloring wool and nylon. The dried leaves of henna contain a significant amount of lawsone, an active substance bestowing them with staining abilities. Environmental biotechnology, a subfield of biotechnology, engages in the production of biomass or renewable energy sources and the elimination of pollutants, utilizing either entire organisms or their by-products. Recent research indicates that henna, owing to its sustainability, abundant production, simplicity of preparation, low cost, and reputation for being safe and ecologically benign, is exceptionally well-suited to participate in the realm of environmental biotechnology. This review navigates through the most recent studies exploring the use of henna and its extracts for related purposes. These encompass a spectrum of applications, including but not limited to nanobiotechnology, fabric dyeing, corrosion resistance, colored solar cells, carbon dots, and new renewable energy exemplified by biofuel and biohydrogen. Furthermore, henna extracts have been deployed to function as antimicrobials and ward off dangerous insects.

## Introduction

It is currently acknowledged that using microorganisms and their byproducts to create nanoparticles from various metals has become a common practice in many laboratories and is considered a green tool. Within this framework, we have made numerous scientific contributions (Zaki et al., 2011; Eltarahony et al., 2018, 2021). A vast majority of these research projects were conducted in labs, with minimal attention given to how these scientific ideas were influencing the industrial realm. Fermenters can only be obtained from accredited research centers and/or biotech businesses since the microbial industry requires fermenters with specialized specifications and skilled hands to protect the environment from microbial contamination After this realization, researchers globally shifted their focus toward developing sustainable, easily accessible alternatives that are both green and environmentally benign. In our research, we used ginger extract to produce AgNPs, CuNPs, and NiNPs nanoparticles, marking it as one of the most significant alternatives that meet these criteria (Kamal et al., 2020).

In the search for plant extracts, henna leaf extract (HL) stands out owing to its unique biochemical composition, making it widely applicable in nanotechnology and other environmental domains. A phytochemical analysis performed by EL-Kamali et al. (2018) showed that the ethanol extract of HL is composed of D-allose (17.61%), lawsone (12.87%), beta-D-glucopyranoside, methyl (12.74%), phytol (10.78%), 1-isobutoxy-1-methoxypropane (9.18%), n-hexadecanoic acid (6.33%), 9,12,15-octadecatrienoic acid (4.44%), squalene (4.06%), and vitamin E (3.60%) as its major phytochemical constituents. Danzarami et al. (2016) conducted a phytochemical screening of henna leaf extracts (HLE) using water, methanol, and ethyl acetate methods and identified the presence of tannins, saponins, flavonoids, alkaloids, glycosides, phenol, and anthraquinones. According to the thinlayer chromatography (TLC) study results, the sample contained approximately nine separate color components with retention factors (Rf) values of 0.2, 0.96, 0.1, 0.3, 0.2, 0.8, 0.1, 0.9, and 0.7. These components are brown, light brown, lemon green, green, dark green, light yellow, and light blue. Notably, while young leaves contain considerably fewer of these compounds than older leaves, the quantity of the active substance, especially lawsone (the coloring pigment), increases in HLE as the plant ages (Danzarami et al., 2016).

**Figure:**
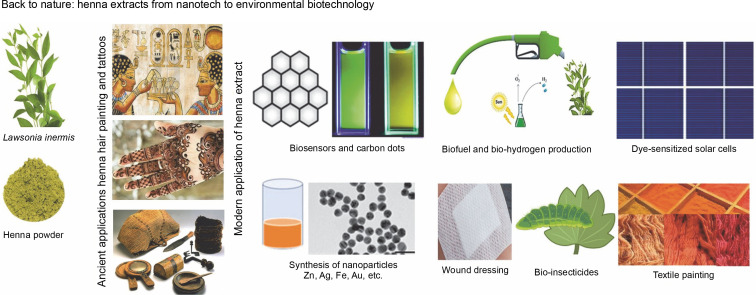


All active chemicals found in henna extracts (HE), whether derived from leaves, stems, seeds, or oils, endow them with potent reductant and stabilizer capabilities for metal nanoparticles, proving beneficial for various applications. In this review, we highlight the most recent and cutting-edge biotech applications that utilize HLE, along with its various applications to provide a clean environment. Recourse to nature and its products has transcended being a scientific luxury, evolving into a necessity aimed at protecting the earth from pollution and global warming.

Recently, several research publications have been presented wherein HE plays a vital role in green nanotechnology, attributable to the ongoing enhancement of laboratory capabilities, requirements, and monitoring and measuring instruments. The formation, shape, size, and diameter of the developed nanoparticles have been confirmed using scanning electron microscopy (SEM), transmission electron microscopy (TEM), X-ray diffraction (XRD), Fourier transform infrared spectroscopy (FTIR), zeta potential, and UV-visible spectroscopy techniques. Tatar and Jha (2022) demonstrated the synthesis of CuONPs utilizing HLE, which consisted of a complex of spherical particles with a size range of 10–80 nm. Dama et al. (2016) demonstrated HLE’s ability to transform silver nitrate into silver nanoparticles (AgNPs). According to Rajan et al. (2018), hexagonal CdSe nanorods, with diameters ranging from 3 to 5 nm and dimensions ranging from 1 to 100 nm, were fabricated using HLE, with CdSe nanorods of lower surface energy being produced along the (0002) orientation by aligning the (112‾0) and (101‾0) planes. Additionally, a peculiarly blue-shifted band gap emerged as a consequence of the nanorods’ impacts on preferred orientation growth.

Using HLE as a capping agent, hexagonal zinc oxide rod nanoparticles (ZnONPs) were formed under alkaline conditions using NaOH and sustained stirring for 2 h (Upadhyaya et al., 2018). Metwally et al. (2022) reported that the green synthesis of ZnO-NPs via the methanolic extract of HLE exhibited outstanding wound-healing efficacy with an undetectable scar, compared to chemically manufactured ZnO-NPs. Additionally, it has been shown that green-synthesized chitosan nanoparticles (Ch-NPs) utilizing HLE increase wound surface area, healing duration, and wound contraction percentage (Metwally et al., 2023). Henna seed extract (HSE) and ultrasound vibrations were utilized to create a spherical and irregular mixture of silver and gold nanoparticles (Au–Ag BNPs), with particle sizes ranging from 15 to 35 nm (Akilandaeaswari and Muthu, 2021). A year earlier, the same authors successfully synthesized AuNPs in spherical, triangular, and polygonal shapes, with diameters ranging from 10 to 55 nm using the same methodological plane (Akilandaeaswari and Muthu, 2020). Apian, one of the active ingredients in HLE dye, was deployed as a reducing and stabilizing agent to generate anisotropic gold and quasi-spherical silver nanoparticles (Kasthuri et al., 2009). Notably, modifying the metal salt to apiin ratio in the reaction medium allowed for meticulous control over the nanoparticles’ size and structure. Spherical iron oxide nanoparticles (IONPs), with an average size of 150–200 nm, were created in the presence of the amino acid L-tyrosine, iron metal, and HLE (Chauhan and Upadhyay, 2019). Silver oxide nanoparticles (Ag_2_O NPs) were generated in an aqueous colloidal condition by combining aqueous silver nitrate with HLE at 75°C with stirring for 1 h (Fayyadh and Alzubaidy, 2021). Without a doubt, the examples given in the study publications regarding the creation of NPs using HE are not exhaustive. Additional models will be covered in the subsequent applications of HE.

## Dye-sensitized solar cells

For more than 50 years, substantial research has been conducted on solar cells. In recent years, dye-sensitized solar cells (DSSCs) have emerged as a more economical, straightforward, and easily fabricated alternative. This electrochemical system generates a semiconductor through the combination of a photosensitive anode and an electrolyte. Aiming to emulate the process of photosynthesis in plants, these naturally pigmented solar cells capture light, storing it as photons in a layer of crystalline nanoparticles (Jasim et al., 2012). The addition of henna color into this field has provided two crucial dimensions: first, it can serve as the primary pigment in solar cells, and second, it can be used to stabilize and reduce minerals for their conversion into biological nanocrystals. In this context, a simple hydrothermal method was utilized to fabricate the working electrode for the ZnO nanorods (Sakthivel and Baskaran, 2014). XRD and SEM methods were employed to explore the crystallinity and morphology of the resultant electrode. Researchers have investigated the impacts of temperature, dye pH, solvent used for dye preparation, and natural dye extracts (beetroot, henna, and amla) on the characteristics of solar cells. It was demonstrated that the efficiency of ZnO nanorod solar cells sensitized with HE surpassed that of solar cells sensitized with beetroot and amla extracts. Esakki et al. (2022) engineered DSSCs using colors derived from henna, applying the solvothermal technique at different pH levels (3–9), and utilizing ZnONPs created with HLE as a photoanode. The ZnONPs achieved the highest efficiency of 0.39% at pH 9, outperforming other solar cells.

Henna flower extract (HFE), rather than HLE, was used by Hao et al. (2014) to create a nanoTiO_2_ solar cell. Their research revealed that the nanoTiO_2_ solar cell had a 400 nm conversion efficiency of 0.15%, an open-circuit voltage of 0.39 V, a short-circuit current density of 0.66 mA cm^2^, a fill factor of 0.60, and an IPCE of 1.7%. Jasim et al. (2012) investigated the optical and photovoltaic characteristics of sensitized nanostructured TiO_2_ with HLE, optimizing conditions for creating the nanocrystalline TiO_2_ layer. They found that the performance of the dye-sensitized photovoltaic cell was contingent upon the choice of redox electrolyte. In a recent study, Hendi et al. (2023) developed DSSCs using dyes extracted from henna and spinach as sensitizers, iodine as electrolytes, and TiO_2_NPs as photoelectrodes. These DSSCs demonstrated fill factors of 0.09 and 0.37, achieving efficiencies of 0.24 and 2.19% for spinach and henna dyes, respectively. Onuigbo et al. (2021) fabricated magnesium-doped TiO_2_ solar cells, dye-sensitizing, and assembling them using methanol extracts from several Western African plants, including henna. The Mg^2+^-TiO nanoparticles were applied to fluorine-doped tin oxide (FTO) glass to operate as the photoanode of the solar cells with an electrolyte. According to their findings, Mgdoped TiO_2_ exhibited the most significant enhancement, increasing by 93% from 0.66 to 1.28 (mA/cm^2^). Furthermore, utilizing magnesium as a doping agent enhanced the photo-generated electron transport but also enhanced the light-harvesting capabilities of the henna dye, thereby boosting the efficiency of light-to-electricity conversion in photovoltaic cells.

## Biofuel and biohydrogen production

Undoubtedly, the persistent escalation in the global population has increased the demand for automobiles of various kinds. Consequently, the imperative to explore sustainable and environmentally friendly alternative fuels has intensified due to the dwindling fossil resources. Biofuel has thus emerged as a notably significant alternative. It is derived from numerous sources such as vegetable oils, animal fats, organic compounds, and even used cooking oil and other waste materials.

Aravind et al. (2021) conducted a study wherein henna essential oil was extracted from seeds through a cold-pressing technique. The extracted oil was subsequently transesterified to yield henna methyl esters. Mixtures, designated as B10, B20, and B30, represented methylated henna oils combined with pure diesel in proportions of 10, 20, and 30% by volume, respectively. These mixtures were tested against a single-cylinder diesel engine running on pure diesel, with engine performance characteristics of BTE, BSFC, and EGT, as well as emissions of CO, HC, and NOx, being observed and compared to those of pure diesel. The results implied that the B20 blend, which reduced the amount of 20% pure diesel fuel, could be utilized in place of plain diesel if preferred.

Khan et al. (2021) employed henna and actinomycete DU-C2 in the production of AgNPs for biohydrogen production. In batch configurations, *Clostridium beijerinckii* was incubated with henna-mediated AgNPs at concentrations of 0, 40, 80, and 120 g/ml for 72 h, resulting in an increase in hydrogen production with a yield of 1.71 mol H2/mol added glucose. The synthesis of AgNPs by DU-C2 impeded bio-H2 production during batch H2 fermentation. During batch fermentation, AgNPs, integrated with both living and deceased cells, were examined to assess their impact on the bacterial culture. While DU-C2 AgNPs precipitated a reduction in bacterial growth, henna-mediated AgNPs amplified H2 generation and increased the amount of bacteria. This enhancement is attributable to their greater ability to stabilize the pH at 6, smaller size, and more potent zeta potential. Furthermore, under such conditions, predominant acids, including butyric and acetic acids, were generated.

## Engines cooling

One of the most crucial operational needs for combustion engines, especially those with high energy and substantial thermal loads, is cooling. One of the most effective strategies for delivering efficient cooling is utilizing nanofluids, primarily due to their extensive exposed surface area. This allows for the downsizing of heat exchangers while boosting the liquid’s heat transfer and distribution coefficients (Siczek et al., 2020). The robust thermophysical characteristics of these nanofluids encompass their behavior amidst various base fluid concentrations and their viscosity, stability, zeta potential, thermal conductivity dispersion, and heat dissipation efficiency. According to a review study by Siczek et al. (2020), copper oxide nanoparticles (CuONPs) stand out as one of the most promising additives for coolant. To utilize nanofluids for heat transmission, a study by Tatar and Jha (2022) employed leaf extracts from neem and henna plants to biosynthesize CuONPs. The target nanoparticles, produced by both extracts, ranged in size from 10 to 80 nm and consisted of spherical particles of complex shape. Physical properties of CuONPs, including their thermal conductivity, viscosity, surface tension, and pH, were identified. The findings indicated that adding CuONPs to water not only accelerated the cooling rate of the water but also enhanced the physical characteristics of the nanofluid, increasing thermal conductivity and viscosity by 10.45% and 10.75%, respectively.

## Microbial and insect biocides

Henna extracts and oils have traditionally been used to treat various infectious disorders, including those caused by *Pseudomonas aeruginosa*, group B *Streptococcus agalactiae*, and *Trichomonas vaginalis*. In the wake of the emergence of antibiotic-resistant bacteria, the use of novel strategies and alternative methods might assist in mitigating future resistance (Bafghi et al., 2022). Research by Mickymaray et al. (2016) has demonstrated that henna exhibits several environmentally beneficial effects, encompassing antibacterial, antifungal, and antiparasitic properties. Given the multitude of advantages that nanotechnology has conferred upon materials — especially regarding the wide range of their applications and notably in contexts of using affordable quantities for environmental remediation and adhering to green and environmentally friendly methodologies in their development. In order to be used as an anti-food-borne microbial pathogen, Ghazy et al. (2023) described the formulation of HE as a nanoemulsion with a droplet size of 90 nm. The formulation was executed using Tween 80 or a blend of Tween 80 and SDS, aided by ultrasonic waves. When tested against *Bacillus cereus*, *Escherichia coli*, and *Pseudomonas aeruginosa*, its antimicrobial activities surpassed penicillin in antifungal activity (*P* < 0.05), and it demonstrated overall enhanced effectiveness against bacteria.

In another study, Hü et al. (2013) electrospun HLE, polyethylene oxide (PEO), and polyvinyl alcohol (PVA) to craft nanofibers, which could potentially serve as antibacterial agents. According to their findings, HLE at 2.793 weight percent in PVA and PEO-based solutions displayed bactericidal effects against *Staphylococcus aureus* and bacteriostatic effects against *E. coli*. The antibacterial activity against both bacterial strains was significantly influenced by the concentrations of HLE.

Rahuman et al. (2022) showcased the efficacy of HLE as a biocide against various bacteria: Gram-positive strains like *Streptococcus* sp., *Streptococcus aureus*, and *Bacillus* sp., and Gram-negative strains including *E. coli*, *Proteus mirabilis*, *Klebsiella pneumoniae*, and *Pseudomonas aeruginosa*. Fayyadh and Alzubaidy (2021) posited that the antibacterial activity of Ag_2_ONPs produced using HLE was more potent against Gram-negative bacteria compared to Gram-positive ones. According to Mohamed (2018), the nontoxicity level of AgNPs generated by HLE makes them applicable for eradicating microbiological pathogens from water and wastewater. Al-Esawi and Al-Musawi (2022) demonstrated that ZnONPs synthesized by HLE exhibited antibacterial efficacy against bacteria like *S. aureus*, *E. coli*, *Proteus mirabilis*, and *Klebsiella pneumonia*. Upadhyaya et al. (2018) discovered that ZnONPs synthesized using HLE outperformed those produced without it in terms of antibacterial activity. Chauhan and Upadhyay (2019) discovered that IONPs manufactured based on henna inhibited pathogenic bacterial strains – *S. aureus* and *Staphylococcus typhimurium* – with an average inhibition area of 1.6 and 1.5 cm, respectively.

Research into henna extracts and metal nanoparticles derived from them has extended into the realms of pesticides and microbicides. Amuthavalli et al. (2021) investigated the antimosquito activity of ZnONPs biosynthesized using HLE. The results of field studies indicated a reduction in mosquito larval and pupal populations over a duration of 24–72 h. In breeding locations, nanoparticles enhanced the predatory efficacy of mosquitofish, *Gambusia affinis*, and copepods. Marimuthu et al. (2012) studied the loculicidal action of synthesized AgNPs against *Pediculus humanus* capitis and *Bovicola ovis* using HLE. The mixtures displaying the most active properties were HLE, 1 mM AgNO_3_ solution, and AgNPs. The outcomes demonstrated that the generated AgNPs had exceptional antiloculicidal activity.

## Biosensors and carbon dots

Quantum dots (QDs) have long been respected for their outstanding electrical and optical features, such as size, tunable emission wavelength, extensive absorption spectrum, composition, photostability, and brightness, alongside a high absorption coefficient and quantum yields reaching up to 85% (Rezaei et al., 2017). The creation of QDs involves the use of semiconductor materials such as selenium, tellurium, and cadmium. However, the preparation of these materials necessitates several steps and are poisonous and expensive (Zuo et al., 2016). Emerging as a recent alternative to QDs, carbon dots (CDs) have emerged with innovative qualities such as excellent photobleaching resistance, robust photoluminescence, venial cytotoxicity, and chemical inertness. Additionally, they possess significant functionalization potential with diverse groups for a range of applications, particularly in biotechnology, as well as remarkable water solubility and stability, among other characteristics. Consequently, hydroxyl, carboxyl, and amino functional groups were found to be present on the CDs’ surface, as per Tripathi et al. (2017).

A simple, free, and one-step hydrothermal synthesis method for CDs was devised by Shahshahanipour et al. (2017), utilizing henna leaf powder as a novel green source. It has been illustrated that henna CDs can serve as a label-free fluorescent probe for the sensitive and specific detection of MTX, a pivotal cancer medication, in human plasma serum and aqueous solutions. The CDs produced were also employed as an antibacterial agent against Gram-positive and Gram-negative infections. The findings revealed that henna CDs could successfully eliminate both Gram-positive and Gram-negative microbes. Summarily, the henna CD methodology offers numerous advantages, including the ability to replenish the carbon source, a simple, cost-effective synthesis that requires no chemical reagents, a one-pot procedure, and the production of notably stable CDs. The henna leaf-derived, blue fluorescence-emitting CDs were doped with Fe^2+^ and were characterized using UV-vis spectroscopy and luminescence. The effect of doping on fluorescence emission was also explored, using quinine sulfate as a reference. Subsequently, their quantum yield was determined. The quantum yield of the henna CDs was found to be 12.37%.

## Heavy metals removal

The surge in industrial and human activities has undeniably elevated the concentration of heavy metals in wastewater. Operations such as battery manufacturing, plating, electroplating, pesticide production, rayon manufacturing, mining, tanning, metal rinse processes, textiles, fluidized bed bioreactors, petrochemical developments, paper production, electrolysis applications, and metal smelting are some of these activities. Consequently, the release of effluent contaminated with heavy metals into the environment poses tangible threats to ecosystems and human health. Particularly concerning is that heavy metals are nonbiodegradable and may cause cancer, their inappropriate concentration in water poses a serious risk to the health of all living things. In light of these challenges, henna has been explored in recent studies as a green and biological method for adsorbing heavy metals from wastewater, according to Qasem et al. (2021).

The ability of henna to remove zinc (Zn II) from aqueous solutions was investigated by Davarnejad and Panahi (2016). Their findings demonstrated that optimal Zn (II) adsorption, to the tune of 79%, was achieved at a pH of 5 and a biomass dose of 0.2 g/l, reaching equilibrium adsorption after 60 min. Remarkably, henna showcased a notable potential for heavy metal adsorption with a Langmuir adsorption capacity (qm) of approximately 76.92 mg/g, indicating its viability as an adsorbent. FTIR analyses substantiated that the henna adsorbent possesses a lot of active functional groups capable of adsorbing metal ions. Furthermore, Davarnejad et al.’s (2018) results suggested that dried HL coupled with chitosan nanoparticles as an adsorbent effectively removed Cr (VI) ions from aqueous solutions.

An exploration by Shanthi and Selvarajan (2013) into carbon derived from henna leaves, used as an adsorbent for Cr (VI) and Cu (II) ions from aqueous solutions, revealed that the maximum percentage of metal removal was attained with an adsorbent dosage of 0.7 g and an initial concentration of 100 ppm metal ions. Notably, the removal percentage for Cu (II) was found to be greater compared to Cr (VI). High absorbance for Cd (II) was noted upon the conjugation of henna with chitosan microparticles in research conducted by Davarnejad and Dastnayi (2019). Shafiee et al. (2018) also scrutinized the removal of Pb (II) ions using henna, investigating the impacts of operational parameters such as pH, contact time, initial metal concentration, and adsorbent dosage under optimal conditions.

In the study conducted by Davarnejad and Panahi (2016), copper (Cu II) was removed from aqueous solutions by henna modified with Fe_3_O_4_ nanoparticles. SEM and XRD analyses of the Fe_3_O_4_ nanoparticles revealed that the modified henna considerably enhanced the stability, coating, and adsorption. In this study, a central composition design (RSM), various parameters, including time, solution pH, initial Cu (II) concentration, and dose adsorbent, were analyzed for their effects on the adsorption process. Notably, the modified henna demonstrated efficacy in extracting copper (II) from wastewater, as evidenced by the difference between initial and final concentrations of Cu. The removal of chromium (VI) (Davarnejad et al., 2018) and cadmium (II) (Davarnejad and Dastnayi, 2019) ions in the presence of chitosan microparticles and henna as adsorbents was studied. These studies employed an RSM design to scrutinize several variables, such as time, initial Cr (VI) or Cd (II) concentrations, henna concentration, number of chitosan microparticles, and pH, to understand their impact on the adsorption process. Intriguingly, the research showed that henna was capable of adsorbing both Cr (VI) and Cd (II) ions from aqueous solutions. This lends credence to the proposition that henna could be a useful approach for eliminating them from the soil.

## Bioremediation of Hazardous organic compounds

Biodegradation has been recognized as a cost-effective, safe, and promising method for the removal or detoxification of hazardous organic compounds (Abd-ElHaleem et al., 2002, 2003). In light of this, Abd-Elaziz et al. (2018) prepared and stabilized AuNPs with HLE, hypothesizing potential applicability for bioremediation processes involving dichlorodiphenyltrichloroethane (DDT). Degradation experiments, conducted at DDT concentrations of either 10 or 20 mg/l, revealed a maximum DDT degradation of 64.1% at 10 mg/l and 77.4% at 20 mg/l after 72 h of incubation. The degradation was determined and analyzed using UV-vis, FTIR, and GC mass spectroscopy. Five peaks, confirming DDT degradation, emerged in the GC mass spectra: dichlorodiphenyldichloroethylene (DDE), dichlorodiphenyldichloroethane (DDD), dichlorodiphenylmethane (DDM), and dichlorophenylethane (DCE). Similarly, Akilandeaswari and Muthu (2021) synthesized a mixture of Au-AgBNPs using HSE and demonstrated, in the presence of NaBH_4_, remarkable catalytic activity for the reduction or degradation of 4-nitrophenol and methyl orange color. It was observed that increasing the nanoparticle dose reduced the time required for breakdown. Additionally, Nandeshwar et al. (2022) employed AgNPs generated using HLE to degrade Azo dyes, specifically Azoblack, Alizarine, Indigo Carmine, and Tartrazine, achieving degradation rates approaching 90%.

Nachimuthu et al. (2022) examined the degradation of a dye mixture using a hybrid technique, which combines photocatalysis with LI-ZnO and LI-ZnO/Fe_2_O_3_ nanorods, and biological treatment using microorganisms. HLE was employed as a capping agent throughout the nanoparticle production. The team discovered that applying photocatalytic treatment before the biological process accelerated decolonization and exhibited deterioration. Consequently, compounds treated with photocatalysis proved to be highly biodegradable by microorganisms in the subsequent biological process. After 5 days of post-treatment with LI-ZnO, Fe_3_O_3_, and H_2_O_2_, the biological degradation process achieved 98% efficacy. Additionally, Ramasamy et al. (2021) explored the photocatalytic degradation of atenolol (ATL) and acetaminophen (ACT) in secondary effluent pharmaceutical residues, utilizing Ag-ZnO produced with HLE. The optimal conditions for the studies were established at 5 mg/l of pollutants, a pH of 8.5, and a catalyst dosage of 1 g/l, resulting in visible light irradiation caused by the Ag-doped photocatalyst.

## Biosurfactant, cement, and mud formulation

Surfactants are ubiquitously utilized in the formulation of emulsions, foams, wetting capacity, cleaning dispersants, and even agents with antioxidant and antibacterial activities (Lukic et al., 2016). Synthetic surfactants are nonbiodegradable, hence they must be replaced by biodegradable surfactants that are low in toxicity or non-toxic and sustainable. Natural surfactants generated from microorganisms and plants are an alternative that has been thoroughly researched and is currently being used commercially by several companies worldwide (Jahan et al., 2016). Particularly, surfactants sourced from plants have gained special interest since, in addition to their usage in ancient communities such as detergents and shampoos the yield in extraction procedures may be higher than that produced for microbial biosurfactants (Du et al., 2020).

Henna has a considerable presence in this field of study. Tavakoli et al. (2020) explored the viability of employing HLE as a natural surfactant and nanoparticle synthesizer for titanium dioxide (TiO_2_), silicon dioxide (SiO_2_), graphene, and a TiO_2_-graphene composite. The primary goal of this study was to minimize the oil–water interfacial tension. Nanoparticles were formulated using the sol-gel process and validated through XRD, FESEM, EDAX, and FTIR assays. Nanosurfactants were stabilized with Tragacanth extract, a natural water-based suspending surfactant, posited as a viable alternative to commercial nanoparticle stabilizers in the oil sector. Interfacial tension (IFT) studies indicated that amplifying the HE content from 0% to 10% diminished the IFT between kerosene and water from 37.23 to 15.24 mN/m. Moreover, introducing 1 wt% TiO_2_ nanoparticles to HE surfactant reduced IFT from 18.43 to 14.57 mN/m. Given this significant reduction in IFT values, the oil recovery factor during EOR operations could be notably augmented. The findings of Tavakoli et al. (2020) demonstrated that TiO_2_ nanosurfactant was as effective as industrial surfactants.

Water saturation and hydrocarbon resource assessment significantly influence the enhancement of oil reservoirs, as highlighted by Kamaei et al. (2019). The cementation factor, identified as an exponent in the Archie equation and determined by the electrical characteristics of the formation, directly impacts the results. Consequently, a small modification in the cementation factor resulted in a large difference in the water saturation and oil reserve predictions. Kamaei et al. (2019) investigated the influence of wettability changes on the cementation factor of carbonate rocks when employing a natural surfactant. The results showed that the rock’s wettability changed at different concentrations of HE (a natural surfactant), ranging from 1 to 10% by weight. Furthermore, the wettability of carbonate pellets reduced from 140.45 to 102.7 mN/m in terms of the contact angle, while the cementation factor for HE-saturated rock decreased from 3.91 to 1.54. All aforementioned studies underscore the vital function that henna and its extracts can potentially fulfill in natural surfactant production as well as in the petroleum sector.

Natural surfactant solutions have been deemed efficient based on their concentrations at the interface and adsorption on rock surfaces. Ghasemi et al. (2019) evaluated the importance of HE as a natural surfactant due to its nonadverse effect on corrosion rates, nontoxicity, and cost-effectiveness. They investigated HLE as a natural substitute for salts and nanoparticles to enhance shale stability. Swelling, immersion, and gravimetric experiments were used to determine the transport of ions and water into the shale. The inhibitory properties of HE and the effect of salts on shale instability were investigated and compared to nanoparticles. The effect of HE on blocking pore throats was studied using pore pressure transmission tests. The results indicated that HLE might reduce the quantity of ions that water can transport into shale. At a concentration of 3% (wt), HLE completely sealed the pore mouths of the shale, outperforming nanoparticles. This investigation confirmed that HLE is an environmentally beneficial shale inhibitor.

The most important short-term approach for preserving the productivity of a crude oil well is the acid treatment of reservoirs. Adding hydrochloric acid (HCl) to tanks presents numerous challenges, including damage to cement columns and insulation, as well as adverse environmental impacts. The reader may be wondering what role HLE plays in addressing these environmental and economic concerns. Aghajafari et al. (2016) developed a modified cement mortar incorporating HLE to resist acid attacks. The parameters of the henna-modified cement were contrasted with those of the reference mortar (0.44 w/c), which was composed merely of cement and water. The cured mortar was also subjected to a 0.1 M hydrochloric acid solution at temperatures ranging from 25 to 55EC. Research findings on the characterization and interaction with hydrochloric acid confirmed the significant protective role of cement slurry, modified with HLE, in improving cement resistance to acid attack and benefiting from it in environmentally appropriate oil well acidification treatments.

Oseh et al. (2019) created henna water-based mud (HWBM), and its efficacy in diminishing cuttings transport efficiency (CTE) was compared to that of bentonite water-based mud (BWBM) and fresh water. Experiments were conducted in a laboratory-scale flow loop, utilizing a 16-foot-long test section constructed from a concentric annulus outer casing (3.5-in. ID) and a static inner drill pipe (2.4-in. ID). Testing occurred at hole angles of 0, 45, 60, 75, and 90°, with annular fluid velocities of 1.1, 1.54, 1.98, and 2.31 ft/s, and a cutting diameter of 1.0 mm. The results showed that HWBM had better rheological and filtration properties compared to BWBM. However, upon heating, the plastic viscosity of BWBM and HWBM reduced by 6.76 and 1.54%, respectively, while their yield points declined by 21 and 4.1%. A hole angle of 45° emerged as the most challenging, demanding careful consideration in drilling mud application.

## Corrosion inhibitor

Key conditions for employing a corrosion inhibitor encompass the efficiency and protection of the target metal, ensuring the inhibitor does not negatively impact the barrier, and maintaining the coating for an extended period. In this context, several researchers have explored the potential of HLE as a corrosion inhibitor. According to the study findings by El-Etre et al. (2005), HLE acted as a corrosion inhibitor for carbon steel, nickel, and zinc in both alkaline and acidic conditions. Their findings demonstrated that the type and qualities of the mediator influenced the degree of inhibition. Notably, the efficacy of zinc was greater in acidic environments than in alkaline or neutral ones. These data supported the idea that HLE functioned as a mixed corrosion inhibitor (El-Etre et al., 2005), demonstrating superior inhibitive efficacy in alkaline, neutral, and acidic conditions for steel, nickel, and nickel alloys. Al-Sehaibani (2000) investigated HLE as a corrosion inhibitor for steel and commercial aluminum in saline, acidic, and alkaline water. The metal exhibited minimal inhibition in the presence of NaCl, while aluminum showed 99.8% efficacy in the presence of NaOH, and mild steel demonstrated 96% efficacy in the presence of HCl.

A mixture of HLE and paint created a barrier with an inhibitory efficiency of 96.6% that increased with rising HLE content, according to research by Nik et al. (2017) on the corrosion of aluminum alloy 5083 in seawater. Chaudhari and Vashi (2016) explored the use of HLE in an acetic acid solution to halt mild steel corrosion. In their study, dynamic polarization, weight loss, and AC resistance were investigated. The results revealed that while the corrosion rate increased with the acid concentration, the corrosion inhibition efficacy improved with the concentration of HLE. Furthermore, HLE was used as a corrosion inhibitor to protect the steel N80 API steel while it was immersed in an acidic mud medium at temperatures between 28 and 75°C. Abdollahi et al. (2021) studied the effects of acid stimulation on reservoir rocks and liquids. Their study validated the chemical adsorption of henna particles on the metal surface using thermodynamic analysis. When henna particles were added to a typical acidic slurry, this inhibitor precipitated an additional 0.96% by weight of sludge, which was the least amount compared to the primary acid and other acidifying additives obtained from a commercial source. The outcomes also showed that HLE makes the samples oily and wet. In summary, the outcomes of these comprehensive investigations suggest that HLE can be reliably utilized in sandstone acidification.

Fouda et al. (2019) investigated HLE as a carbon steel (C-steel) corrosion inhibitor in a 1 M hydrochloric acid solution. The following phenomena were estimated: mass loss, dynamic effective polarization, alternating current resistance, and electrochemical frequency modulation. According to the findings, HLE adsorption influenced both cathodic and anodic interactions. Furthermore, increasing the extract concentration improves the inhibitor’s activity.

Meanwhile, Khoshkhou et al. (2018) explored the resistance to corrosion of low-carbon steel, utilizing HLE as an inhibitor, integrated with a TMSM–PMMA coating. Through the construction of the TMSM hybrid cell and the creation of carbon steel substrates via a dipcoating process that involved hydrolysis and densification. The findings obtained with various dosages of HLE revealed the inhibitory efficacy of the coating containing 3% inhibitor in 0.1 M HCl. The polarization tests also supported El-Etre et al. (2005) assertion that HLE operates as a mixed inhibitor.

## Dyeing and finishing of fabrics

Chemical dyes are currently used to color textiles because of their inexpensive cost and extensive color pallet. Petroleum byproducts are the principal source of them. Researchers in many scientific schools who are interested in dying fabrics have been increasingly interested in the trend of identifying natural replacements for chemical dyes This growing interest stems from stringent environmental regulations that categorize numerous petroleum products as poisonous and hazardous to both the environment and human health. Consequently, several organic alternatives, sourced from microorganisms, plants, or animals, have been developed. Among these, a dye extracted from the leaves of the henna plant, known as HLE, is perceived to be superior. HLE is environmentally friendly, quick, and meets consistent technical requirements (Alebeid et al., 2015). To extract natural dye from HLE, Ali et al. (2009) enhanced alkaline conditions for cotton dyeing. Their study compared the results of dyeing without mordants to those of premordanting and postmordanting with alum and iron. The findings showed that investigations comparing synthetic and natural dyes suggested that natural henna dye had a good chance of cooperating with synthetic dye.

According to the findings of Aalipourmohammadi et al. (2020) obtained enhanced self-cleaning properties, efficient antibacterial activities, and UV-blocking for wool fabrics when employing HLE as both a coloring and a capping/reducing agent for the antimicrobial nanoparticles ZnONP. Another study by Alebeid et al. (2015) evaluated HLE and TiO_2_ nanosols for cotton fabric dyeing and functional finishing, finding that samples treated with TiO_2_ and dyed with HLE significantly improved in UV protection and antibacterial efficiency without affecting color depth and tensile strength.

Yadav et al. (2019) investigated linen cloth naturally dyed using a combination of henna and copper sulfate. Their findings suggested that henna-dyed linen could be positioned in the market as a green multifunctional textile material, offering washable color and efficient protection against bacteria, UV light, and free radicals, with the multipurpose features persisting even after 20 washes. Devj et al. (2019) combined bactericidal henna with chitosan to enhance the antibacterial properties of wool materials. This resulted in chitosan-treated wool fabrics exhibiting enhanced antibacterial properties and color uptake. Sharma et al. (2009) sought to reuse henna dyeing wastewater in the coloring and multifunctional finishing of linen fabric in their study. The findings demonstrated that chitosan-treated wool fabrics had improved antibacterial properties and color uptake. Sharma et al. (2009) sought to reuse henna dyeing wastewater in the coloring and multifunctional finishing of linen fabric in their study. The results demonstrated commendable color metrics, antioxidant capacities, and UV protection. Analytical data of the wastewater – evaluating chemical oxygen demand (BOD), conductivity, total dissolved solids (TDS), pH, and bath redox potential – indicated that nearly no mordant salt remained in the exhaust bath after three dyeing cycles.

Ragheb et al. (2017) developed a unique henna natural dye with a nanoscale size of about 100 nm using an ultrasonic stirrer method. Their research investigated the colorfastness characteristics of printed natural fabrics, including silk, wool, and cotton, in both the presence and absence of a mordant. The data showed that manufactured nanohenna dye performed better than the original dye combined with an alum mordant when applied to silk and cotton fibers. Beyond natural fabrics, henna can also dye synthetic materials. Research conducted by Arain et al. (2021) offered a green methodology, utilizing microwave technology to mordant and dye polyester cloth with natural henna dye. Lemons were used as a natural mordant in microwaves, ensuring a truly eco-friendly and green dyeing procedure. The authors verified that the microwave approach significantly reduced the mordanting and dyeing time by 60–65%, while also enhancing color fixation.

## Future perspectives

Plant extracts, widely employed in traditional medicine to treat various ailments, can be derived from numerous parts of the plant, such as leaves, fruits, stems, and roots. Before administering them to patients, it is imperative to conduct relevant biochemical and clinical tests and investigations to ensure patient safety. Moreover, these extracts find frequent use in animal feed, biocides, cosmetics, and medicines. Numerous extracts have been validated by rational scientific research as effective insecticides, antifungals, parasiticides as well as significant antioxidants and immune boosters. The utility of these plants lies in their sustainability, low to nonexistent toxicity, producing a lot of mass and requiring little irrigation water and agricultural fertilizers. Nonetheless, enhanced collaboration between corporate and academic research is required to accelerate the development of these extracts in order to fulfill the pressing need for them in the health, environmental, and industrial sectors. Many researchers have recently focused their efforts on utilizing HE in various applications such as nanoparticle fabrication, photovoltaic solar cells, carbon dots, biofuels, biohydrogen, surfactants, and other applications. The mechanism by which the metal transitions from microparticles to nanoparticles in henna extracts, however, warrants further investigation. Since HE comprises a complex mix of active chemicals, various chelating agents might significantly influence the volume of nanoparticle synthesis. Consequently, it could be scientifically preferable to isolate the bioactive chemicals and test them individually to determine whether these compounds might be utilized independently or in a synergistic combination. Using statistical analysis to compare several outcomes in the gathered study may provide useful information in this regard. Working on ways to make the extract to the smallest possible scale (nanoscale), and coming up with creative ways to build the tools needed to make it without the usage of ultrasound or microwaves. The product will change as a result, improving its qualities and enabling it to be used in a variety of industrial applications.

On the other hand, recent advancements in genetic engineering could play a pivotal role in cultivating henna with chemical components that amplify the production of biomolecules needed for creating nanoparticles, biosurfactants, anticorrosives, or even the enzymes necessary for the biodegradation of harmful cyclic chemicals. Alternatively, enhancing the carbon content throughout the entire henna plant might render it a superior absorber for heavy metals from water and sewage. Additionally, this demands comprehensive research on how to breed or genetically modify plants to generate more proteins, enzymes, and other biomolecules required by numerous sectors that promote health and the environment. To harness phytochemicals and maximize their potential in all manufacturing processes for plant extracts, particularly in henna plants, continuous scientific research is essential. This is attributed to the fact that these chemicals are more cost-effective, abundantly producible, and sustainable than other biological sources.

It has been highlighted that although active polymers in HE are employed across numerous industries, including nanotechnology, antimicrobials, and medication delivery, recent scientific research concerning these polymers is notably sparse. Despite these benefits and the aforementioned advantages, comprehensive research remains imperative in this domain. Undoubtedly, exhaustive research will be crucial to translate these studies from the laboratory to the factory to ensure optimal output. This has been made easier by various sophisticated statistical software programs, many of which are available online at no cost, that consider all influential parameters such as pH, temperature, production time, optimal concentrations, and other factors.

Additionally, exploring separation and extraction technologies for more future industrial and commercial applications in a pure form – free from the inconveniences of their widespread use, which might impact public health and the environment – is also deserving of research funding and teamwork. A pressing need exists to conduct thorough investigations to identify optimal procedures for executing the extraction and drying processes in less time than is currently required and to strive towards substituting safe or natural solvents for those used in extraction, which are categorically listed as being harmful to human health. Some physical techniques, in my opinion, might currently be the most environmentally friendly.

Interestingly, I could not locate any information in the published literature regarding the use of HE as an antipollution agent in marine environments without adversely affecting the flora and animals – whether it is effective in removing heavy metals and oil spills, as well as reducing BOD and other pollutants. Genetic engineering could significantly contribute to the development of henna plants that yield a variety of natural colors, safe for human health and potentially usable for painting walls and wood, and even as a base for iron doors and facades. This correlates with how HE, inherently rich in coloring chemicals, has been utilized for millennia in hair coloring, human tattooing, and dyeing fabrics of various types.

## Conflicts of interest/Competing interests

The author declares that there is no conflict of interest.
